# Supplementary material for: Genome-Wide Association Study of HIV Whole Genome Sequences Validated using Drug Resistance
Source: PLoS One. 2016 Sep 27;11(9):e0163746. doi: 10.1371/journal.pone.0163746 (PMC5038937; doi:10.1371/journal.pone.0163746)
Supplement: S1 File — Additional figures and tables for secondary analyses of the data. (DOCX) [file pone.0163746.s001.docx]

Supplementary Materials

Table A: Results of secondary analyses of top SNPs in original GWAS

| **Drug** | **SNP** | **Effect allele** | **OR** | **p-value** | **OR only those treated for:** | | **Length of treatment** | | **Correcting for other treatments** | | **Correcting for PCs** | | **Correcting for Relatedness** | |
| --- | --- | --- | --- | --- | --- | --- | --- | --- | --- | --- | --- | --- | --- | --- |
|  |  |  |  |  | **6+ months** | **12+ months** | **Beta** | **p-value** | **OR** | **p value** | **OR** | **p-value** | **OR** | **p-value** |
| Nevirapine | 3078G | G | 5.19 | 4.77E-10 | 5.13 | 5.16 | -34.9 | 0.6 | 4.38 | 6.09E-05 | 3.51 | 0.00025 | 5.123 | 7.23E-10 |
| Stavudine | 2739A | A | 0.084 | 5.38E-06 | 0.086 | 0.091 | 86.3* | 0.07* | 0.109 | 0.0011 | 0.08 | 0.002 | 0.07875 | 2.46E-05 |
| Tenofovir | 1063A | G | 1.79 | 2.42E-05 | 1.83 | 1.82 | 17.4 | 0.6 | 1.66 | 0.0025 | 1.64 | 0.01 | 1.833 | 1.40E-05 |
|  | 2730G | G | 6.44 | 1.67E-14 | 6.95 | 7.5 | 64 | 0.031 | 4.58 | 1.61E-08 | 6.73 | 9.40E-10 | 6.504 | 6.20E-14 |
|  | 2738G | G | 2.89 | 1.45E-05 | 3.15 | 3.27 | 58.4 | 0.13 | 2.71 | 0.00029 | 3.28 | 0.00063 | 2.878 | 1.57E-05 |
|  | 2852A | A | 1.72 | 6.19E-05 | 1.7 | 1.77 | 13.2 | 0.67 | 1.72 | 0.0021 | 1.43 | 0.068 | 1.744 | 4.81E-05 |
|  | 2880T | T | 5.77 | 1.80E-05 | 6.36 | 6.93 | 101 | 0.007 | 4.16 | 0.0025 | 3.65 | 0.0039 | 5.753 | 1.86E-05 |
| Zidovudine | 2745G | G | 3.11 | 2.94E-07 | 3.11 | 3.01 | 81.5 | 0.34 | 4.79 | 2.13E-08 | 3.77 | 9.62E-05 | 3.139 | 3.26E-07 |

*results relate to length of treatment for tenofovir, as stavudine association a proxy for tenofovir DR due to negatively correlated phenotypes.

Table B: P values for known drug resistance amino acids, showing an excess of nominally significant associations.

| **Drug and Gene** | **Amino acid** | **BP1** | **BP2** | **BP3** | **P1** | **P2** | **P3** |
| --- | --- | --- | --- | --- | --- | --- | --- |
| **Stavudine** | 41 | 2657 | 2658 | 2659 | 0.29 | 4.40E-16 | 0.09 |
| RT | 65 | 2729 | 2730 | 2731 | NA | NA | NA |
|  | 67 | 2735 | 2736 | 2737 | 0.09 | 0.99 | 0.91 |
|  | 70 | 2744 | 2745 | 2746 | 0.64 | 0.02 | 0.52 |
|  | 210 | 3164 | 3165 | 3166 | 0.61 | 0.99 | 0.47 |
|  | 215 | 3179 | 3180 | 3181 | 0.08 | 0.09 | 0.87 |
|  | 219 | 3191 | 3192 | 3193 | 0.11 | 0.36 | 0.12 |
| **Tenofovir** | 65 | 2729 | 2730 | 2731 | NA | 1.67E-14 | 0.41 |
| RT | 70 | 2744 | 2745 | 2746 | 0.01 | 0.77 | 0.22 |
| **Zidovudine** | 41 | 2657 | 2658 | 2659 | 0.07 | NA | NA |
| RT | 67 | 2735 | 2736 | 2737 | 7.56E-04 | 0.31 | 0.72 |
|  | 70 | 2744 | 2745 | 2746 | NA | 2.94E-07 | 0.45 |
| **Efavirenz** | 100 | 2834 | 2835 | 2836 | NA | NA | 0.46 |
| RT | 101 | 2837 | 2838 | 2839 | 0.03 | 0.07 | 0.89 |
|  | 103 | 2843 | 2844 | 2845 | NA | 0.04 | 3.03E-04 |
|  | 106 | 2852 | 2853 | 2854 | 2.60E-04 | 0.01 | 0.97 |
|  | 108 | 2858 | 2859 | 2860 | 0.53 | NA | 0.15 |
|  | 181 | 3077 | 3078 | 3079 | NA | 3.93E-11 | 0.35 |
|  | 188 | 3098 | 3099 | 3100 | 0.14 | NA | 0.35 |
|  | 190 | 3104 | 3105 | 3106 | 0.99 | 0.41 | 0.91 |
|  | 225 | 3209 | 3210 | 3211 | NA | 0.03 | 0.89 |
|  | 230 | 3224 | 3225 | 3226 | 0.98 | NA | NA |
| **Nevirapine** | 100 | 2834 | 2835 | 2836 | 0.68 | NA | 0.20 |
| RT | 101 | 2837 | 2838 | 2839 | 0.02 | 0.18 | 0.54 |
|  | 103 | 2843 | 2844 | 2845 | NA | 0.88 | 0.91 |
|  | 106 | 2852 | 2853 | 2854 | 4.48E-04 | 0.03 | 0.83 |
|  | 108 | 2858 | 2859 | 2860 | 0.89 | NA | 0.18 |
|  | 181 | 3077 | 3078 | 3079 | NA | 4.77E-10 | 0.68 |
|  | 188 | 3098 | 3099 | 3100 | 0.07 | 0.05 | 0.59 |
|  | 190 | 3104 | 3105 | 3106 | 0.99 | 0.09 | 0.35 |
|  | 230 | 3224 | 3225 | 3226 | 0.25 | NA | NA |
| **Lopinavir** | 10 | 2267 | 2268 | 2269 | 0.82 | NA | NA |
| PR | 20 | 2297 | 2298 | 2299 | NA | 0.26 | 0.67 |
|  | 24 | 2309 | 2310 | 2311 | 0.63 | NA | NA |
|  | 32 | 2333 | 2334 | 2335 | NA | NA | 1.00 |
|  | 33 | 2336 | 2337 | 2338 | NA | NA | NA |
|  | 46 | 2375 | 2376 | 2377 | NA | NA | NA |
|  | 47 | 2378 | 2379 | 2380 | NA | NA | NA |
|  | 50 | 2387 | 2388 | 2389 | NA | NA | NA |
|  | 53 | 2396 | 2397 | 2398 | NA | NA | NA |
|  | 54 | 2399 | 2400 | 2401 | NA | NA | 0.65 |
|  | 63 | 2426 | 2427 | 2428 | 0.77 | 0.04 | 0.77 |
|  | 71 | 2450 | 2451 | 2452 | NA | NA | 0.50 |
|  | 73 | 2456 | 2457 | 2458 | NA | NA | NA |
|  | 76 | 2465 | 2466 | 2467 | 0.17 | NA | 0.98 |
|  | 82 | 2483 | 2484 | 2485 | 0.62 | NA | NA |
|  | 84 | 2489 | 2490 | 2491 | NA | NA | NA |
|  | 90 | 2507 | 2508 | 2509 | NA | NA | NA |

Table C: Significant epistatic interactions between significantly associated SNPs with tenofovir exposure. Results generated using plink’s –epistasis option and restricting to genome-wide significant SNPs only.

| **SNP1** | **SNP2** | **Effect** | **p-value** |
| --- | --- | --- | --- |
| 2730G | 2738G | 6.22 | 0.013 |
| 2738G | 2739A | 12.77 | 0.00035 |
| 2738G | 2880T | 10.98 | 0.00092 |
| 2739A | 2880T | 4.57 | 0.033 |

Table D: Significant associations in original analysis using sequence AF411967 as a reference compared to in a second reference sequence (AY228557).

|  |  | **AF411967** | |  |  |  | **AY228557** | |  |  |  |
| --- | --- | --- | --- | --- | --- | --- | --- | --- | --- | --- | --- |
| **Drug** | **SNP** | **BP** | **A1** | **Reference** | **OR** | **p-value** | **BP** | **A1** | **Reference** | **OR** | **p-value** |
| Nevirapine | 3078G | 3078 | G | A | 5.2 | 4.77E-10 | 3115 | G | A | 4.685 | 9.00E-08 |
| Stavudine | 2739A | 2739 | A | G | 0.08 | 5.38E-06 | 2776 | A | G | 0.1432 | 5.70E-06 |
| Tenofovir | 1063A | 1093 | G | A | 1.79 | 2.42E-05 | 1076 | G | A | 1.619 | 0.000998 |
|  | 2730G | 2730 | G | A | 6.44 | 1.67E-14 | 2767 | G | A | 7.727 | 8.15E-14 |
|  | 2738G | 2738 | G | A | 2.89 | 1.45E-05 | 2775 | G | A | 2.875 | 0.0001 |
|  | 2852A | 2852 | A | G | 1.72 | 6.19E-05 | 2889 | A | G | 1.707 | 4.23E-04 |
|  | 2880T | 2880 | T | A | 5.77 | 1.80E-05 | 2817 | T | A | 4.635 | 1.26E-05 |
| Zidovudine | 2745G | 2745 | G | A | 3.11 | 2.94E-07 | 2732 | A | C | 3.692 | 0.00032 |

Fig A: Distribution of allele frequencies for variants where minor allele frequency is greater or equal to 1%

Fig B: Average non-missingness across the genome in all samples for each SNP, with sequenced segments of genome shown for reference (PAN1-4)


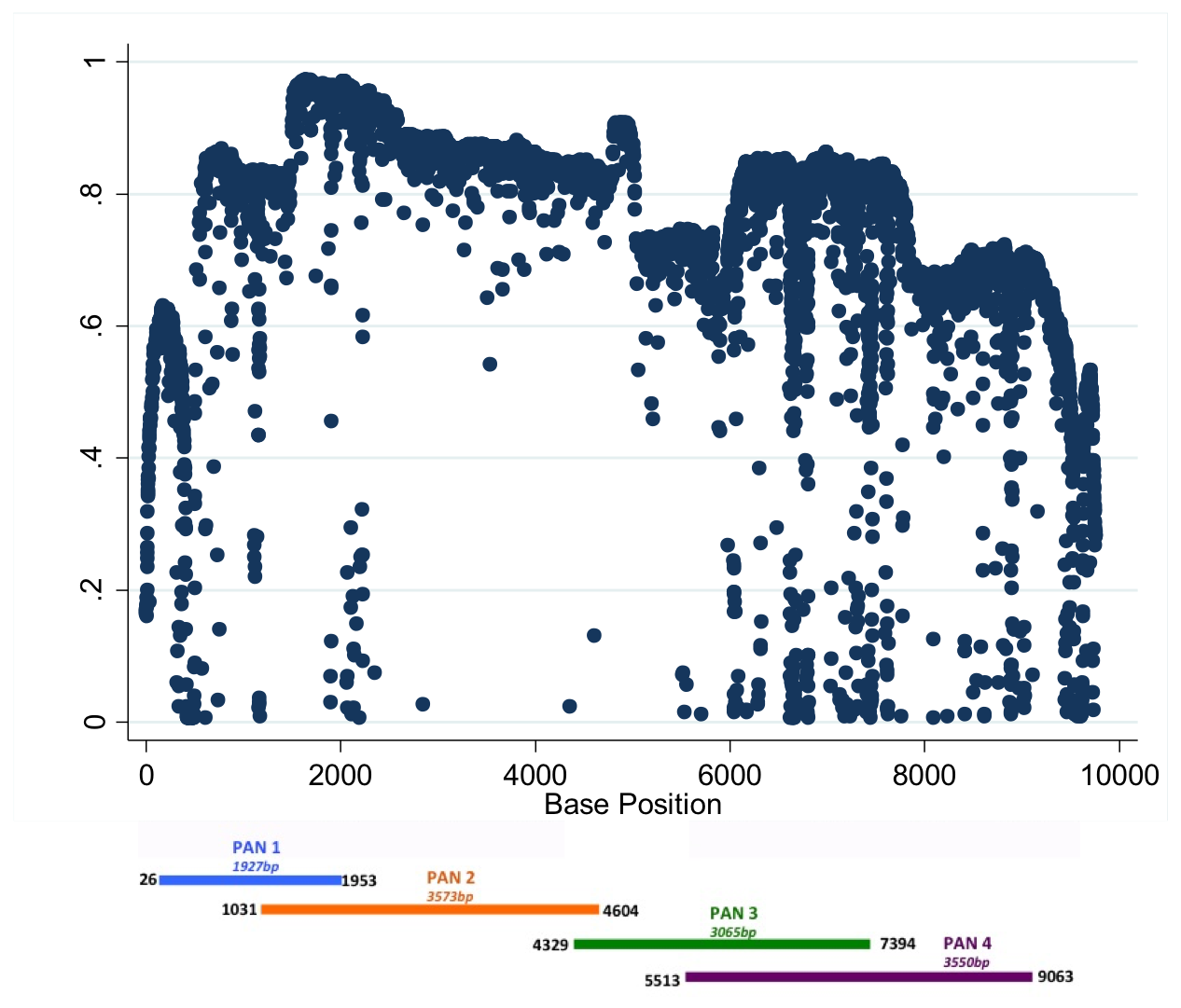


Fig C: Manhattan plot comparing HIV sequences that were exposed to zidovudine to those that were not. The reference line at p=7E-5 is the line for genome wide significance. Dashed grey lines on genomic locations refer to borders of genes (black dashed refer to GAG, Pol and ENV).

Fig D: Manhattan plot comparing HIV sequences that were exposed to nevirapine to those that were not. The reference line at p=7E-5 is the line for genome wide significance. Dashed grey lines on genomic locations refer to borders of genes (black dashed refer to GAG, Pol and ENV).

Fig E: Manhattan plot comparing HIV sequences that were exposed to stavudine to those that were not. The reference line at p=7E-5 is the line for genome wide significance. Dashed grey lines on genomic locations refer to borders of genes (black dashed refer to GAG, Pol and ENV).

Fig F: Manhattan plot comparing HIV sequences that were exposed to lopinavir to those that were not. The reference line at p=7E-5 is the line for genome wide significance. Dashed grey lines on genomic locations refer to borders of genes (black dashed refer to GAG, Pol and ENV).

Fig G: Manhattan plot comparing HIV sequences that were exposed to efavirenz to those that were not. The reference line at p=7E-5 is the line for genome wide significance. Dashed grey lines on genomic locations refer to borders of genes (black dashed refer to GAG, Pol and ENV).

Fig H: QQ plot of observed –log p values for each SNPs association with efavirenz (y-axis) vs distribution of –log p values from permuted phenotype (x-axis). Dashed line is reference for y=x.

Fig I: QQ plot of observed –log p values for each SNPs association with nevirapine (y-axis) vs distribution of –log p values from permuted phenotype (x-axis). Dashed line is reference for y=x.

Fig J: QQ plot of observed –log p values for each SNPs association with lopinavir (y-axis) vs distribution of –log p values from permuted phenotype (x-axis). Dashed line is reference for y=x.

Fig K: QQ plot of observed –log p values for each SNPs association with stavudine (y-axis) vs distribution of –log p values from permuted phenotype (x-axis). Dashed line is reference for y=x.

Fig L: QQ plot of observed –log p values for each SNPs association with tenofovir (y-axis) vs distribution of –log p values from permuted phenotype (x-axis). Dashed line is reference for y=x.

Fig M: QQ plot of observed –log p values for each SNPs association with zidovudine (y-axis) vs distribution of –log p values from permuted phenotype (x-axis). Dashed line is reference for y=x.
